# The impact of COVID-19 on microRNA and CD marker expression in AML patients

**DOI:** 10.1038/s41598-024-64775-1

**Published:** 2024-06-20

**Authors:** Rastee H. Saeed, Zirak Faqe Ahmed Abdulrahman, Dara K. Mohammad

**Affiliations:** 1 Department of Biology, College of Education, Salahaddin University-Erbil, Erbil, Kurdistan Region Iraq; 2 College of Agricultural Engineering Sciences, Salahaddin University-Erbil, Erbil, Kurdistan Region Iraq; 3 Center for Hematology and Regenerative Medicine (HERM), Department of Medicine Huddinge, Karolinska Institutet, 141 83 Stockholm, Sweden

**Keywords:** AML, COVID-19, microRNA, CD biomarker, Gene mutations, Cancer, Biochemistry, Cancer, Cell biology, Genetics, Immunology, Molecular biology, Biomarkers, Diseases

## Abstract

Acute myeloid leukaemia (AML) is an aggressive leukaemia characterised by uncontrolled blast cell proliferation. miRNAs and Clusters of Differentiation (CD) molecules play essential roles in AML progression. This study aims to investigate the effect of COVID-19 on the expression of circulating miRNA and CD molecules in AML. This cross-sectional study recruited 32 AML patients and 20 controls. Blood samples were collected and analysed using molecular cytogenetic, miRNA/mRNA expression, and flow cytometry techniques. The expression of miRNAs varied significantly between patients with AML and control individuals. The co-expression of these miRNAs was higher (P < 0.05), indicating that the presence of one miRNA led to increased expression of other miRNAs. A differential correlation was observed between miRNAs and CD markers. Additionally, miRNA 16, miRNA 21, and miRNA 221 showed significant downregulation (P < 0.05 and P < 0.01, respectively) in AML patients with COVID-19 infection compared to those without a disease. Interestingly, this study identified a higher expression level (P < 0.01) of miRNA 137 as a novel biomarker for AML patients. Moreover, the expression of miRNA 137 showed a high correlation (P < 0.05) with most of the CD markers examined in this study and FISH features data. Furthermore, a strong correlation (P < 0.01) was observed between CD markers and miRNA among AML patients with positive and negative COVID-19 infection. These data demonstrated that COVID-19 contributed to increased expression of microRNAs in AML patients. MicroRNA 137 was identified as a novel microRNA that exhibited significant differences between patients and healthy individuals, highlighting its role in AML pathogenesis.

## Introduction

Acute myeloid leukaemia (AML) is an aggressive form of leukaemia that is commonly diagnosed in older individuals (≥ 60 years)^1^. It is distinguished by the uncontrolled proliferation of immature blast cells in the peripheral blood and bone marrow, leading to impaired red blood cell production and the failure of bone marrow function. This condition can give rise to severe complications, as abnormal cells disrupt the average production of healthy blood cells, impairing the immune system and resulting in anaemia and bleeding^2^. AML is the most ubiquitous type of leukaemia that occurs in adults, and its global incidence is about 162/1 cases per million, which increases with age. Although AML’s outcome has improved in terms of treatment, its overall survival rate is still under 50%^3^. AML is a highly heterogeneous disease characterised by a wide range of genetic and epigenetic alterations that play an essential role in developing the disorder. The disease’s complexity and heterogeneity make understanding its underlying molecular mechanisms crucial for developing targeted therapies and improving patient outcomes^4^.

MicroRNAs are short non-coding RNA molecules (about 21–25 nucleotides in length) that control gene expression at the post-transcriptional stage by binding to the 3′-untranslated regions (3′-UTRs) of target messenger RNAs (mRNAs)^3,5^. This interaction results in mRNA degradation or the suppression of translation, which modifies the expression of proteins involved in various cellular processes like proliferation, differentiation, and apoptosis^6,7^. Numerous clinical illnesses, including AML, have been linked to the aetiology of miRNA expression dysregulation^8,9^. Overall, the Wnt signalling pathway, melanogenesis, and other cancer-associated signalling pathways may be associated with the development of AML due to differences in their expression of pleomorphic adenoma gene 1 (PLAG1), frizzled class receptor 3 (FZD3), annexin A2 (ANXA2), has-microRNA-155, and has-microRNa-192, may play crucial roles in the oncogenesis of AML. hsa-miR-155, has-miR-192, ANXA2, FZD3, and PLAG1^10^.

Cell surface proteins known as Clusters of Differentiation (CD) molecules play a crucial role in the characterising and functioning of immune cells. They serve as markers to classify different leukaemia subtypes and evaluate the presence of minimal residual disease (MRD) in patients with AML^11^. Emerging research indicates that miRNAs can regulate the expression of CD molecules, affecting the behaviour of immune cells and ultimately impacting the advancement and prognosis of AML^12,13^. For example, the percentage of CD11c + tumour-associated macrophages and their activation state are decreased, suggesting one of the potential pathways shifting in the tumour's cytokine milieu towards an M2/Th2 phenotype^14^.

Severe acute respiratory syndrome coronavirus 2 (SARS-CoV-2) has significantly impacted global health, leading to millions of infections and deaths worldwide^15^. Besides the direct effects on respiratory health, recent evidence suggests that COVID-19 can also affect the immune system and interact with various diseases, including AML. AML patients are often immunocompromised due to the disease or its treatment, and COVID-19-induced immunomodulation may have implicated the clinical outcome of AML^16,17^. Additionally, SARS-CoV-2 can affect the expression of miRNAs and their target genes, potentially contributing to disease severity and complications^18,19^. It has been suggested that COVID-19’s frequently expressed plasma microRNA-16 functions as a defence against lung damage following infection^20^. In a lipopolysaccharide (LPS)-induced damage cell model, Cai et al. found that overexpression of micrRNA-16 reduces TNF-α and IL-6, thereby inhibiting systemic inflammatory responses and lowering acute lung injury^21^. By directly inhibiting PI3-K and decreasing inflammation, micrRNA-16 overexpression can influence TNF-α production and NF-κB activation^22^.

Growing evidence suggests that miRNAs have emerged as promising biomarkers in leukaemia, enabling improved classification of subtypes, prognostic stratification, and prediction of treatment response^23^. However, the interplay between COVID-19, circulating miRNAs, and cell surface markers protein in AML patients with different cytogenetic risk profiles remains largely unexplored. Therefore, it was necessary to conduct this study to address the lack of knowledge regarding the impact of COVID-19 on the expression and correlation of circulating miRNAs and CD molecules in AML patients with different cytogenetic risk profiles. In this study, we found that miRNA expression varied considerably between AML patients and controls, and the presence of one miRNA boosted the expression of other miRNAs. Additionally, we found variable connections between miRNAs and CD markers. Moreover, miRNA 16, miRNA 21, and miRNA 221 exhibited substantial downregulation in AML patients with COVID-19 infection compared to those without a disease. This study found miRNA 137 expression as a unique biomarker for AML patients. Furthermore, a high correlation was identified between CD markers and miRNA in AML patients with positive and negative COVID-19 infection.

## Materials and methods

### Sample collection and molecular analysis

In this paper, we conducted a case–control study, recruiting participants with AML as cases and healthy individuals as controls, to investigate the association between microRNA, CD markers expression, and COVID-19 development. This study employed an experimental design, randomly assigning participants to AML or control group.

#### Blood samples

In this study, blood samples were collected from two groups of individuals: the first group was patients with newly diagnosed AML, and the second group was healthy donors. These samples were collected at Nanakali Hospital for Blood Diseases and Cancer in Erbil, Iraq, over eight months after obtaining written informed consent from all participants. All the necessary ethical considerations were taken into account during the sample collection. Fifty-two samples were collected, consisting of 32 patients diagnosed with AML and 20 healthy controls. All AML patients were analysed using a combination of morphological and flow cytometric analyses with a thorough review of the patient’s medical history and a complete clinical examination. Blood samples were collected in tubes containing EDTA, one tube designated for mRNA and microRNA studies, and another containing sodium-heparin for molecular cytogenetic analysis (FISH). Following collection, blood samples were immediately transferred to a laboratory for storage at − 80 °C and the study protocol was approved by the regional Ethics. Nasopharyngeal and oropharyngeal swabs were collected from patients for COVID-19 PCR testing and placed in a viral transport medium. All experimental protocols were authorised and approved by the Human Ethics Committee of the College of Science, Salahaddin University-Erbil (Approval Ref No: 1770 on 20210614).

#### Cytogenetic analysis

For the molecular cytogenetic analysis, Fluorescence in situ hybridisation (FISH) was performed using a multi-probe panel to detect inv(16)(p13;q22), t(15;17)(q22;q21), t(8;21)(q22;q22), and 11q2.3 abnormalities, mixed lineage leukaemia (MLL) involving translocations, and abnormalities (deletions or trisomy) of chromosomes 5, 7, 8, 9, 11, 13, or Y. A minimum of 200 interphase nuclei were examined for each FISH analysis.

#### Preparation and quantification of mRNA and miRNA

MicroRNA and mRNA extraction were performed using the FavorPrepTM MicroRNA Isolation Kit and FavorPrepTM mRNA Isolation Kit (both from FAVORGEN BIOTECH CORP, Taiwan), following the manufacturer’s instructions. Total microRNA and mRNA quality were assessed using a NanoDrop spectrometer (Biometrics, OneDrop TOUCH Pro/Lite Micro-Volume Spectrophotometer, Wilmington, USA), measuring the 260/280 nm wavelength ratio. cDNA synthesis of MicroRNAs (using reverse transcriptase with microRNA-specific stem-loop primers) and mRNA was conducted using the AddScript cDNA Synthesis Kit (add bio), as per the manufacturer’s instructions, targeting 10 specific MicroRNAs (MicroRNA15a, MicroRNA16, MicroRNA21, MicroRNA125a, MicroRNA125b, MicroRNA137, MicroRNA155, MicroRNA192, MicroRNA221, MicroRNA497). Specific RT primers were used to detect each microRNA, while oligo (dT) primers were used with mRNA for housekeeping gene detection (Table 1). PCR reactions were performed, and qRT-PCR was conducted to determine microRNA expression using the SYBR green method following the manufacturer’s guidelines (AddScript RT-PCR SYBR Master (2 × conc.), Korea). A universal reverse primer and a specific forward primer for each microRNA were employed in the qRT-PCR reaction (Table 1); all primer sequences used in this study were provided in each PCR tube and held a total volume of 25 µl for the RT-PCR process. The components were as follows: 2 µl of cDNA template RT + /RT−/water/patient sample/control; 9 µl of PCR water; 0.5 µl of each of the 100 pmol/µl forward and reverse primers; and 13 µl of SYBR Green PCR Master Mix as described in^24^.

**Table 1:** List of RT-PCR, microRNA forward and universal primer sequences.

miRNA	Primer sequence	Primer type
miRNA-15a	5′-GTCGTATCCAGTGCAGGGTCCGAGGTATTCGCACTGGATACGACCACAAAC-3	RT-PCR
miRNA-16	5′-GTCGTATCCAGTGCAGGGTCCGAGGTATTCGCACTGGATACGACCGCCAA-3	
miRNA-21	5′-GTCGTATCCAGTGCAGGGTCCGAGGTATTCGCACTGGATACGACTCAACA-3	
miRNA-125a	5′-GTCGTATCCAGTGCAGGGTCCGAGGTATTCGCACTGGATACGACTCACAG-3	
miRNA-125b	5′-GTCGTATCCAGTGCAGGGTCCGAGGTATTCGCACTGGATACGAC-3	
miRNA-137	5′-GTCGTATCCAGTGCAGGGTCCGAGGTATTCGCACTGGATACGACATCCAC-3	
miRNA-155	5′-GTCGTATCCAGTGCAGGGTCCGAGGTATTCGCACTGGATACGACCCCTAT-3	
miRNA-192	5′-GTCGTATCCAGTGCAGGGTCCGAGGTATTCGCACTGGATACGACCACTGG-3	
miRNA-221	5′-GTCGTATCCAGTGCAGGGTCCGAGGTATTCGCACTGGATACGACGAAACCCATCACAA-3	
miRNA-497	5′-GTCGTATCCAGTGCAGGGTCCGAGGTATTCGCACTGGATACGACAAACCA-3	
miRNA-15a	5′-GCGGCTAGCAGCACATAATGG-3	Forward
miRNA-16	5′-TAGCAGCACGTAAATATTGGCG-3	
miRNA-21	5′-GCCCGCTAGCTTATCAGACTGATG-3	
miRNA-125a	5′-TCCCTGAGACCCTTTAACCT-3	
miRNA-125b	5′-CGTCCCTGAGACCCTAACTT-3	
miRNA-137	5′-GTGACGGGTATTCTTGGGT-3	
miRNA-155	5′-CTCAGACTCGGTTAATGCTAATCGTGATAGG-3	
miRNA-192	5′-CTGACCTATGAATTGACAGCCA-3	
miRNA-221	5′-GTTGGTGGGAGCTACATTGTCTGC-3	
miRNA-497	5′-TCGGGCAGCAGCACACTGTG-3	
All miRNAs	5′-GTGCAGGGTCCGAGGT-3′	Universal reverse
β-actin	Forward 5′-ACTCGTCATACTC CTGCT-3′	Housekeeping gene
	Reverse 5′-GAAACTACCTTCAACTCC-3′	

### Flow cytometry immunophenotyping

Immunophenotyping was conducted using multicolour flow cytometry on bone marrow aspirate and peripheral blood samples. 15,000 events for the CD45-gating strategy were employed to plot and identify the immunophenotype of the blast cells. An acute leukaemia flow panel consisting of 28 antibodies was carried out to screen and differentiate the lymphocyte subpopulation (Supplementary Table 1). Data were acquired on a BD FACSCanto II flow cytometer (BD Biosciences) and analysed using FlowJo software version 10.9.0 (TreeStar Inc., San Francisco, CA, USA).

### Diagnosis of COVID-19 samples

Nasopharyngeal swabs were taken from patients and healthy control and Sansure Novel Coronavirus (2019-nCoV) Nucleic Acid Diagnostic Kit (PCR-Fluorescence Probing; Hunan, China: Sansure Biotech Co., Ltd.) was used for detecting COVID-19, which targeted two COVID-19 genes, namely, the ORF1ab and N genes. Quant Studio 5 (Applied Biosystems; Waltham, MA: Thermo Fisher Scientific) was used for genome amplification. There was a valid internal control (IC) which is < 40 cycle threshold (Ct) value, and a sample was reported to test positive for COVID-19 if any of the two genes, ORF 1ab (Fam) and N gene (ROX) or both genes showed Ct value less than 40.

### Limitations of the study

It is essential to recognize several limitations that may influence the interpretation of our results. Firstly, while the sample size of our study cohort is sufficient for detecting significant trends, it may limit the generalizability of our findings to broader populations. Larger sample sizes would enhance the robustness of our conclusions and allow for more comprehensive subgroup analyses. In our study, we were limited in contributing a larger sample because individuals with a history of disability, psychological, mental, or other chronic diseases and who refused to sign the consent form or were unwilling to participate were excluded from the study. Secondly, our study design may be susceptible to potential biases inherent in retrospective studies. For instance, selection bias could arise due to the non-random sampling of participants, particularly concerning recruiting individuals with a history of COVID-19. Additionally, information bias may occur if there are inaccuracies or inconsistencies in data collection and interpretation related to patients' medical histories and clinical characteristics. Although efforts were made to mitigate these biases through rigorous data collection and analysis procedures, their presence cannot be entirely ruled out. Therefore, prospective studies with longitudinal follow-up would provide more conclusive evidence regarding the causal links between these factors.

### Statistical analyses

Statistical tests were performed using NCSS 2021 (version 21.0.3) and Graph Pad Prism statistical software (version 9.01, Graph Pad, San Diego, CA, USA). The 2^−∆∆Ct^ method was applied to determine the relative quantity for each microRNA^25^, and the result was normalised using β-actin as an endogenous control for variations in cDNA levels. Additionally, the D'Agostino-Pearson test, Shapiro–Wilk normality test, and Kolmogorov–Smirnov test was used to determine whether the data were normally distributed. For comparing the values before and after treatment, a t-test was performed for normally distributed data and presented as means ± SEM; the Wilcoxon test was applied if the data were not normally distributed and presented as median (range). Kruskal–Walli’s test was performed to compare groups. P values < 0.05 were considered statistically significant for all analyses.

## Results

### Clinical assessment of AML patients and PBMCs characterisation

**Figure 1:**
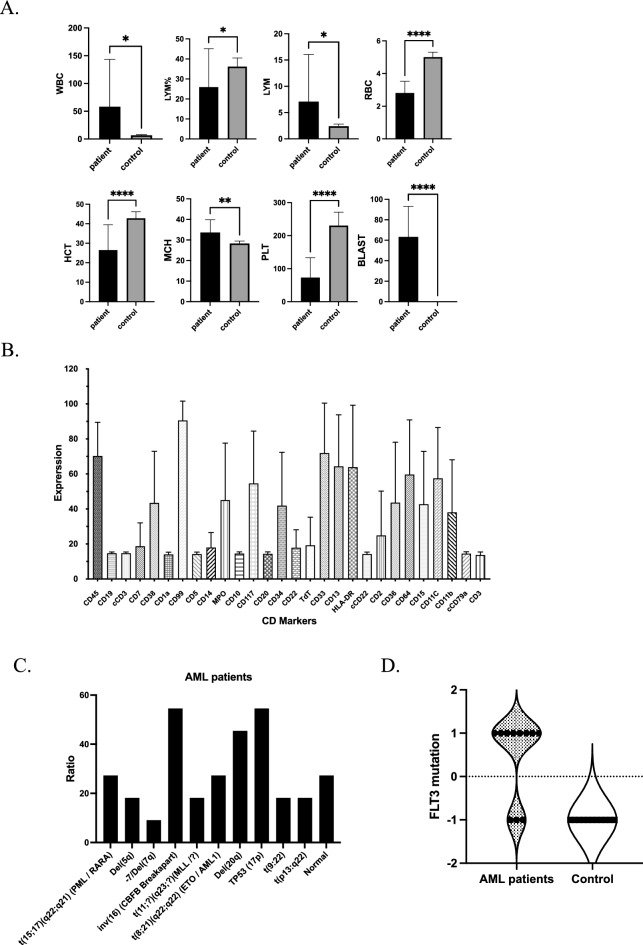
Clinical evaluation of AML patients and characterisation of PBMCs with signature marker detection. (**A**) Complete Blood Count (CBC) Parameters in AML patients and healthy donors. (**B**) The expression of a cluster of differentiation CD markers in AML patients. (**C**) Fluorescence in Situ Hybridization (FISH) probe and genetic abnormalities ratios in AML patients. (**D**) Analysis of FLT3 mutation frequency in AML patients and healthy controls. The data were presented as means of parameter values. *CBC* Complete blood count, *WBC* white blood cell, *LYM%* lymphocyte percentage, *LYM* lymphocyte count, *RBC* red blood cell, *HCT* haematocrit, *MCH* mean cell haemoglobin, *PLT* platelet count, *Statistically significant at 0.05 level.

Figure 1A shows some haematological parameters [Complete Blood Count (CBC)] of AML patients and healthy individuals. The examination of these parameters showed that two groups of people under investigation, AML patients and healthy people, have significant differences (P ≤ 0.05) in the mean of WBC, LYM%, LYM, RBC, HCT, MCH, PLT, and BLAST. The mean of WBC, LYM, MCH, and BLAST levels was higher in AML patients compared to the healthy group; in contrast, the mean of LYM%, RBC, HCT, and PLT levels was higher in the control group compared to the patient’s group (Fig. 1A). Additionally, no differences were observed in the mean parameters of MCV, MCHC, MPV, GRAN, RDW%, RDWa, PDW%, MID%, MID, PCT, LPCR, and PLCC levels between patients and the control group (Supplementary Fig. 1). Interestingly, the flow cytometry data displayed high expression of CD99, CD38, CD33, CD45, CD13, HLA-DR, CD64, MPO, CD117, CD34, CD36, CD11b, and CD11c markers; in contrast low expression of CD19, CD3, CD7, CD1a, CD5, CD14, Cd10, CD20, CD22, and CD79a were observed (Fig. 1B).

Moreover, Fluorescence in situ hybridisation (FISH) was used to identify chromosomal abnormalities in AML patients. According to the FISH data, a higher ratio of inv(16) (p13; q22), TP 53 (17P), del(20q), t(15;17) (q22;q21) and t(8;21)(q22;q22) abnormalities were detected among the AML patients (Fig. 1C). Furthermore, a high frequency of FLT3 mutations was only observed in the AML patients’ group (Fig. 1D).

### MicroRNA expression levels and their correlation with CD markers

**Figure 2:**
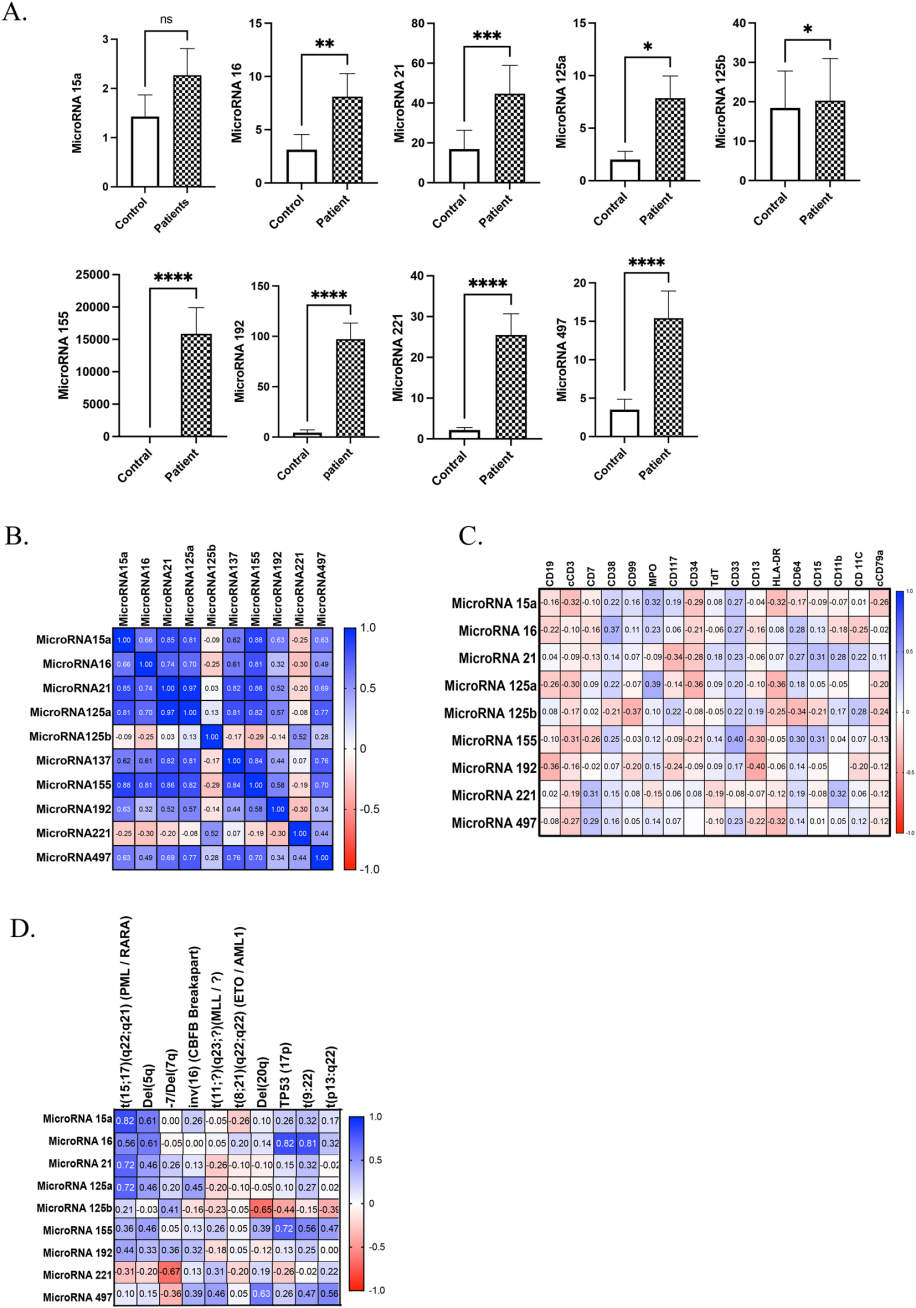
Assessment of microRNA expression and its association with CD markers. (**A**) Differential microRNA expression between AML patients and healthy controls. (**B**) Spearman correlation coefficient analysis of microRNA expression levels, examining the associations and relationships between different microRNAs. (**C**) Spearman correlation coefficient analysis of microRNA expression levels with CD Markers. (**D**) Spearman correlation coefficient between each microRNA with FISH and genetic abnormalities ratios in AML patients.

MicroRNAs can potentially be exploited for clinical diagnosis, prognosis, and cancer therapy, as evidenced by identifying miRNAs linked to chromosomal alterations and clinical outcomes of various leukaemia subtypes. In this study, we tested the expression of 9 MicroRNAs in AML patients and control subjects, as shown in Fig. 2A. Interestingly, a significant upregulation of MicroRNA16, MicroRNA21, MicroRNA125a, MicroRNA125b, MicroRNA155, MicroRNA192, MicroRNA221, and MicroRNA497 was noted in AML patients compared to the control group (P ≤ 0.05) (Fig. 2A). While the expression of MicroRNA15a was slightly higher in AML patients than in healthy individuals, it was statistically insignificant (Fig. 2A). Additionally, we wanted to see if there was any correlation between the above-identified microRNAs; therefore, we performed the Spearman correlation coefficient on the microRNAs. Interestingly, we found that MicroRNA15a, MicroRNA16, MicroRNA21, MicroRNA125a, MicroRNA137, MicroRNA155, MicroRNA192, and MicroRNA497 had a positive correlation with each other, which means the higher expression of one microRNA, associated with increased expression of different microRNAs (Fig. 2B). In contrast, MicroRNA125b and MicroRNA221 expression was negatively connected with other microRNAs, which means the upregulation of these two microRNAs led to the downregulation of other microRNAs (Fig. 2B).

Moreover, we hypothesised that microRNA expression correlations with CD markers could be helpful in early cancer development based on observing distinct biomolecular networks linked to normal and malignant states. In this study, we observed a relative association between microRNA expression and CD markers, where, in some cases, the associations between miRNAs and CD markers were positive (Fig. 2C). In contrast, in other cases, microRNA expression was inversely correlated with CD markers. For example, as shown in (Fig. 2C and Supplementary Fig. 2), miRNA155 positively connected with CD33 expression; in contrast, miRNA192 expression was negatively associated with CD13 expression. Furthermore, the correlation of microRNA expression was also investigated with the progression of fluorescence in situ hybridisation (FISH) probes in AML patients. Interestingly, we found a strong correlation between microRNA expression and FISH data, indicating a higher association of these microRNAs in the increased chromosomal abnormalities in AML patients (Fig. 2D and Supplementary Fig. 3). Also, we demonstrated no significant difference between CD markers in people who were positive or negative for COVID-19 (Supplementary Fig. 4).

### COVID-19 modulates the expression of microRNA 16, 21 and 221

**Figure 3:**
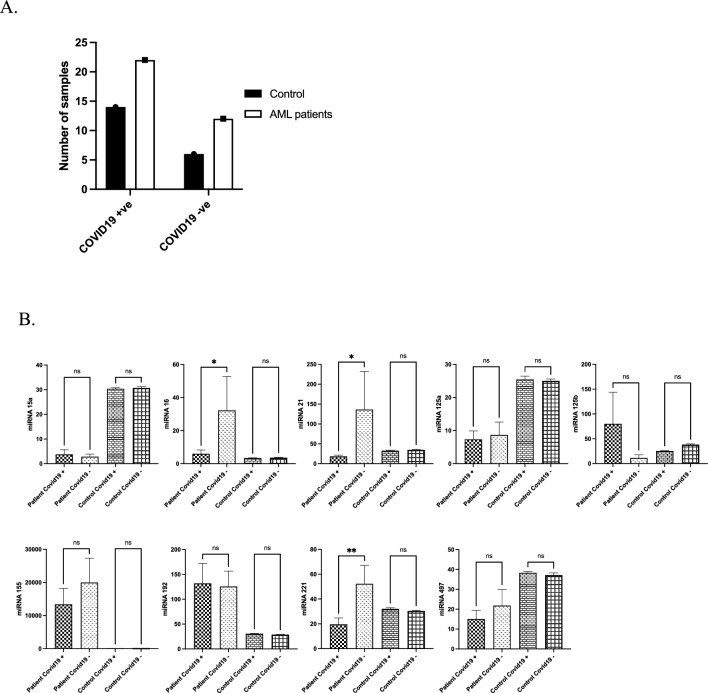
Analysis of microRNA expression levels in AML patients concerning COVID-19. (**A**) Sample size of COVID-19 in AML patients and healthy control. (**B**) microRNA expression patterns in AML and its association with COVID-19.

During the COVID-19 pandemic, we started collecting blood samples from AML patients and healthy donors to test whether COVID-19 impacts microRNA expression. Therefore, we collected samples from 22 AML patients who were COVID-19 positive and 14 healthy donors who were COVID-19 positive (Fig. 3A). Notably, we observed a significant downregulation of microRNA 16, microRNA 21 and microRNA 221 in AML patients positive for COVID-19 (P ≤ 0.05), whereas COVID-19 infection led to a remarkable upregulation of microRNA 125b (Fig. 3B). At the same time, no significant effects on the other microRNAs were identified in AML patients infected with COVID-19 (Fig. 3B). Interestingly, in healthy donors with COVID-19 infection, no significant differences were seen in microRNA expression (Supplementary Fig. 5B).

### COVID-19 influences the association between microRNAs and CD markers

**Figure 4:**
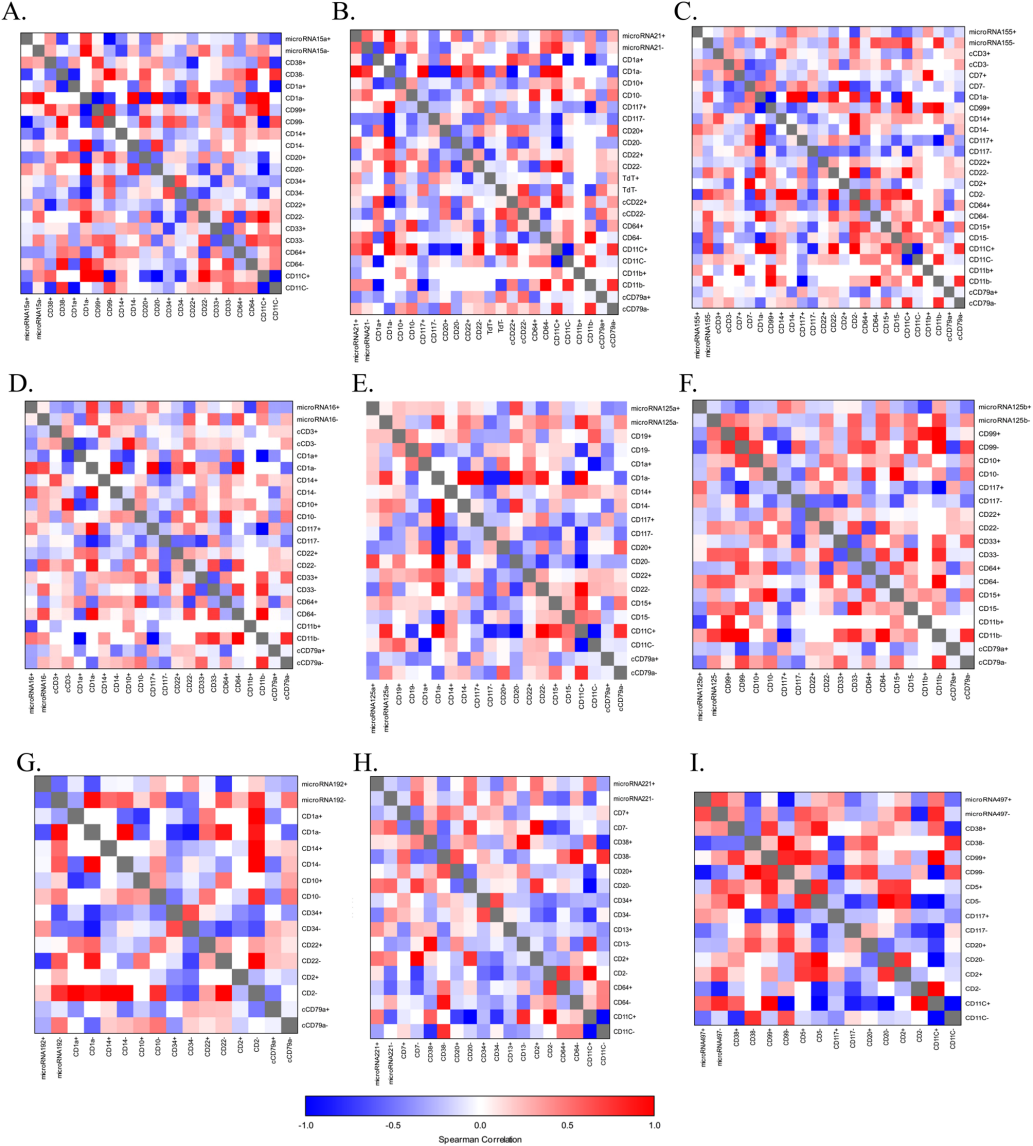
Spearman correlation coefficient analysis between microRNA expression levels and CD markers expression levels in individuals categorised with COVID-19 positive or negative.

Gene expression correlations could be helpful in the early detection of cancer development based on observing distinct biomolecular networks linked to normal and malignant states. In this study, we wanted to investigate the impact of COVID-19 on the correlation between the expression level of each microRNA and CD marker linked during AML progression and could be used for cancer diagnosis. Essentially, the association of microRNA expression with CD markers significantly interfered in AML patients infected with COVID-19 compared to non-infected patients (Fig. 4 and Table 2). Interestingly, we found 9-fold and 7.5-fold changes in microRNA15 correlation with CD1a and CD22 after COVID-19 infection. On the other hand, the correlation of microRNA 16 and microRNA 21 expression with CD1a and CD11b was dramatically changed by 8-fold, 11-fold, and 7-fold, respectively, in COVID-19-positive AML patients. Additionally, the association of microRNA 125b with CD99 and CD11b was strongly affected in AML patients infected by COVID-19. Moreover, the link between microRNA155, microRNA192, and CD1a was remarkably disturbed by ninefold in COVID-19-positive AML patients. Furthermore, COVID-19 infection led to a tenfold increase in the correlation between microRNA221 and microRNA497 with CD2 marker (Fig. 4 and Table 2).

**Table 2:** Fold changes among microRNAs with CD markers in accordance with COVID-19 and Non-COVID-19 AML patients.

Correlation	CD markers	COVID19		P-value +Ve	P-value −Ve	Fold change	Correlation	CD markers	COVID19		P-value +Ve	P-value −Ve	Fold change
		+Ve	−Ve						+Ve	−Ve			
miRNA 15a	CD38	0.3715	− 0.045	0.005	0.0054	3.5	miRNA 137	CD19	0.2101	− 0.3162	0.3821	0.9999	5
	CD1a	0	0.9487	0.9999	0.9999	9		CD1a	0.2739	0.9487	0.9999	0.9999	7
	CD99	0.039	− 0.3	0.0001	0.0001	3		CD14	0.0665	0.4832	0.0558	0.9999	4
	CD14	− 0.4407	0.2352	0.9999	0.9999	6		CD22	− 0.1406	0.6274	0.0704	0.9999	7
	CD20	− 0.4878	0.1293	0.5967	0.9999	5.5		CD33	− 0.1663	0.3727	0.0001	0.0001	4
	CD34	0.0492	− 0.36	0.0004	0.0019	3		CD64	0.2297	0.607	0.0001	0.0001	4
	CD22	− 0.291	0.5488	0.5967	0.9999	7.5		CD11c	− 0.4286	0.2689	0.0001	0.0026	6
	CD33	− 0.0786	0.4374	0.0004	0.0001	4		DC11b	0	0.7	0.9999	0.0629	7
	CD64	− 0.3897	0.3468	0.0001	0.0001	6		cCD79a	− 0.2713	0.3777	0.0944	0.9999	5
	CD11c	− 0.4538	0.0844	0.0001	0.0011	4	miRNA 155	CD3	− 0.0968	0.3453	0.0001	0.0001	4
miRNA 16	cCD3	− 0.3218	0.233	0.7253	0.9999	3		CD7	0.3677	− 0.3162	0.0001	0.0001	6
	CD1a	− 0.2292	0.6325	0.9999	0.9999	8		CD1a	− 0.3651	0.6325	0.0001	0.2454	9
	CD14	− 0.2226	0.3117	0.9999	0.9999	5		CD14	− 0.3579	0.3429	0.0001	0.001	6
	CD10	− 0.2519	0.2946	0.6756	0.9999	4		CD117	0.2347	− 0.2597	0.3506	0.9999	4
	CD117	0.4519	− 0.21	0.0001	0.0207	6		CD22	− 0.1047	0.5455	0.0001	0.0001	6
	CD22	− 0.1977	0.6001	0.8677	0.9999	7		CD2	0	0.6761	0.0001	0.1273	6
	CD33	0.1712	0.5364	0.0001	0.0011	4		CD64	0.1723	0.6386	0.0431	0.9999	5
	CD64	0.0121	0.6491	0.0001	0.0005	6		CD15	− 0.1105	0.5388	0.0001	0.0011	4
	CD11b	− 0.7071	0.4	0.9999	0.8004	11		CD11c	− 0.5021	0.1681	0.9999	0.9999	6
	cCD79a	− 0.3253	0.2506	0.9999	0.9999	5		CD11b	0.3536	0.8	0.0098	0.9999	5
miRNA 21	CD1a	0.0917	0.7379	0.9999	0.9999	7		cCD79a	− 0.2782	0.2842	0.0001	0.0001	4
	CD10	− 0.34	0.5534	0.9999	0.9999	5	miRNA 192	CD1a	− 0.0913	0.9487	0.0106	0.9999	9
	CD117	− 0.001	− 0.47	0.5215	0.9999	4		CD14	− 0.0468	0.5923	0.0044	0.0682	6
	CD22	− 0.4793	0.3	0.9999	0.9999	7		CD10	− 0.1545	0.4603	0.0019	0.006	5
	TDT	0.3488	− 0.014	0.9999	0.9999	4		CD34	− 0.0108	− 0.5003	0.9999	0.9999	5
	cCD22	0.0936	− 0.524	0.9999	0.9999	6		CD22	− 0.03	0.6001	0.0335	0.0059	6
	CD64	− 0.0332	0.594	0.9999	0.0077	5		CD2	0	0.7775	0.0164	0.9999	7
	CD11c	0.5272	0.1814	0.9999	0.7442	4		cCD79a	− 0.2314	0.4937	0.0006	0.0027	7
	CD11b	− 0.3536	0.4	0.9999	0.9999	7	miRNA 221	CD7	− 0.2868	0.5741	0.9999	0.9999	8
	CD79a	− 0.4756	0.1049	0.9999	0.9999	5		CD38	0.11	− 0.206	0.9999	0.9999	3
miRNA 125a	CD19	0.2247	− 0.213	0.9999	0.9999	4		CD20	0.0235	0.5446	0.9999	0.9999	5
	CD1a	0.1833	0.7379	0.9999	0.9999	6		CD34	− 0.1545	0.3771	0.9999	0.9999	5
	CD14	− 0.032	0.4861	0.1118	0.9999	4		CD13	0.3909	− 0.3706	0.0009	0.9999	7
	CD117	0.0922	− 0.526	0.0001	0.0001	6		CD2	0.5	− 0.5071	0.9999	0.9999	10
	CD20	− 0.4256	0.0597	0.0436	0.9999	5		CD64	− 0.1905	0.2842	0.4651	0.9999	4
	CD22	− 0.1528	0.4665	0.1102	0.9999	5		CD11c	0.4686	0	0.1635	0.9999	4
	CD15	0.126	− 0.227	0.0001	0.0001	3	miRNA 497	CD38	0.3068	− 0.5002	0.6249	0.583	8
	CD11c	− 0.0753	0.3814	0.0001	0.0001	4		CD99	0.039	− 0.5	0.0001	0.0045	5
	cCD79a	− 0.4359	0.3978	0.1989	0.9999	7		CD5	− 0.1881	0.4472	0.9999	0.9999	6
miRNA 125b	CD99	− 0.5477	0.4	0.0001	0.0014	9		CD117	0.3642	− 0.287	0.0005	0.0701	4
	CD10	− 0.2157	0.2946	0.9999	0.9999	4		CD20	− 0.2457	0.2178	0.9999	0.9999	4
	CD117	0.5166	− 0.196	0.0004	0.0185	6		CD2	0.2005	− 0.8452	0.9999	0.9999	10
	CD22	− 0.1603	0.4364	0.9999	0.9999	5		CD11c	0.4937	− 0.1513	0.0061	0.3616	6
	CD33	0.0519	0.5818	0.0001	0.0008	5							
	CD64	− 0.272	0.18	0.006	0.0003	3							
	CD15	− 0.2277	0.5096	0.2495	0.0537	7							
	CD11b	− 0.7071	0.7	0.9999	0.9999	14							
	cCD79a	− 0.2056	0.3964	0.9999	0.9999	5							

### MicroRNA137 as a novel biomarker for AML patients

**Figure 5:**
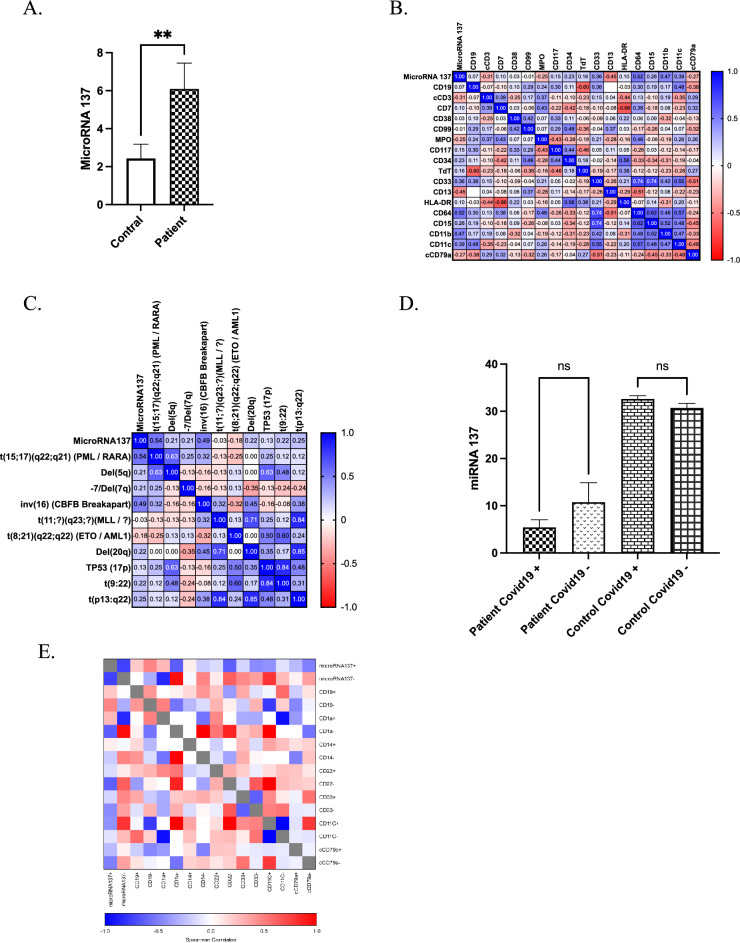
Identification of MicroRNA137 as a novel biomarker for AML patients. (**A**) Expression level of miRNA137 in AML patients and healthy control. (**B**) Spearman correlation coefficient of miRNA137 with CD markers. (**C**) Spearman correlation coefficient of microRNA137 with FISH probes. (**D**) Expression levels of miRNA137 in COVID-19 and non-COVID-19 individuals. (**E**) Spearman correlation coefficient of microRN137A with CD markers in COVID-19 positive or negative.

MicroRNA137 can function as an oncogene that promotes cancer depending on their target genes and functions involved in the initiation and progression of cancers. Some of these target genes include DNMT1 (DNA Methyltransferase 1), FLT3 (FMS-Like Tyrosine Kinase 3), and MLL (Mixed-Lineage Leukaemia). Next, we wanted to examine the expression of microRNA137 in AML patients. Interestingly, we found that microRNA137 was significantly upregulated in AML patients (P ≤ 0.05) and emerged as a novel circulating biomarker for early prediction and development of AML patients (Fig. 5A). Additionally, a higher association was observed between microRNA137 expression and CD64 and CD11 markers in AML patients (Fig. 5B). Moreover, limited research explicitly links microRNA137 to the genetic subtypes of AML, acute promyelocytic leukaemia (APL). Here, we observed that microRNA137 was strongly correlated with t(15;17)(q22;q21)(PML/RARA) and inv(16)(CBFB Breakapart) chromosomal abnormality involved in specific subtypes of acute myeloid leukaemia (AML) (Fig. 5C). Surprisingly, microRNA137 expression was not affected in AML patients infected with COVID-19 compared to non-COVID-19 AML patients (Fig. 5D). However, a slight downregulation of microRNA137 level was observed in COVID-19 positive AML patients (Fig. 5D). Furthermore, the correlation between microRNA137 expression and CD markers among people who were positive for COVID-19 was negatively regulated (Fig. 5E). We also noticed a remarkable change towards a negative correlation of microRNA137 expression with CD1a, CD11c and CD79a in COVID-19-positive AML patients (Fig. 5E).

## Discussion

This study aimed to investigate the expression of microRNAs in leukaemia patients and their association with CD markers. Our results showed significant differences in the expression of microRNAs between AML patients and individuals in the control group. A strong correlation between microRNAs was observed, indicating that the presence of one microRNA led to increased expression of other microRNAs. Additionally, we found that MicroRNA16, MicroRNA21, and MicroRNA221 expression levels exhibited significant differences between AML patients with a history of COVID-19 infection and individuals without an infection history. Moreover, significant fold changes were observed between microRNA expression levels, and the CD markers among AML patients were positive for COVID-19. Finally, the upregulation of MicroRNA137 in AML patients was identified as a novel diagnostic biomarker that may aid in diagnosing, prognosis, and assessing treatment response for AML patients.

The diagnosis and classification of AML require a meticulous approach, as the World Health Organization (WHO) advised, encompassing a comprehensive blend of clinical history, morphological evaluation, cytogenetic/molecular genetic analysis, and immunophenotyping. Nonetheless, the absence of cytogenetic/molecular analyses in numerous healthcare facilities underscores the utility of cytomorphological evaluation supplemented by immunophenotyping, which proves adequate for expedited diagnosis and classification of AML (de Morais et al., 2022).

MicroRNAs are often deregulated in the cancerous process of body cells, and some of them enhance tumorigenesis and cancer progression by increasing tumour growth, angiogenesis, invasion, and immune evasion. The expression level of microRNAs in cancer can be used to predict patient prognosis and clinical response to treatment^26,27^. Since most of the microRNAs are intracellular, circulating microRNAs have been found in different body fluids and are identified as new cancer biomarkers. This study showed that the expression of microRNAs in AML patients increased significantly, and their role in the prognosis of AML disease was well established, similar to other studies^28,29^.

AML patients often have standard karyotypes^30^. They are a heterogeneous group from the molecular point of view, and these molecular differences are related to the prognosis of the disease. Therefore, it is crucial to identify new molecular markers and validate their promising future in treating the disease^31^. One of these essential markers is FLT3. It has been determined that it can be introduced as a valuable marker for diagnosing AML. In this study, we showed that the frequency of FLT3 mutation in AML patients was higher than in the control group and functions as an essential marker in the diagnosis and prognosis of AML disease; these results are consistent with the previous studies^32–35^. Fluorescence in situ hybridisation (FISH) diagnosis was used to detect chromosomal abnormalities in patients with AML, and based on the results of this study, FISH was able to show chromosomal abnormalities and disorders in patients with sufficient accuracy. Previous studies have investigated that the diagnostic strategies of FISH in diagnosing AML can be used as an efficient and powerful tool compared with other methods^36^. Additionally, FISH can be used as one of the standard methods for detecting microRNA expression, which was utilised in this study and effectively demonstrated the expression of microRNAs. Other studies have also shown the reliability of FISH as a method for detecting microRNA expression levels, which is consistent with the present study’s findings. This technique has consistently proven its effectiveness in accurately assessing and quantifying the expression of microRNAs, confirming the results obtained in this research^37,38^.

Recent investigations have unveiled a variety of microRNAs that are dysregulated in AML and play roles in disease pathogenesis. Among these miRNAs are hsa-miR-16, hsa-miR-497, and hsa-miR-125, which have been implicated in regulating NRAS gene expression and cellular processes relevant to cancer development and progression (Hussen et al., 2021, Kipkeeva et al., 2022).

The BCR gene is a key player in the pathogenesis of several cancers, including AML. It encodes a protein regulating cell growth, differentiation, and survival. Dysregulation of the BCR gene, often through chromosomal translocations, can lead to the formation of oncogenic fusion proteins that drive leukemogenesis (Boucher et al., 2023). In the context of AML, aberrant expression of microRNAs has been implicated in disease progression. Specifically, miRNAs such as hsa-miR-16, hsa-miR-497, and hsa-miR-125 have been identified as potential regulators of gene expression and cellular processes relevant to cancer development. These miRNAs may target the BCR gene, influencing its expression levels and activity. Dysregulation of hsa-miR-16, hsa-miR-497, and hsa-miR-125 could thus contribute to aberrant BCR signaling and AML pathogenesis (Szczepanek, 2020). Understanding the regulatory interactions between genes and these miRNAs in AML patients is crucial for elucidating the molecular mechanisms underlying the disease and identifying potential therapeutic targets. Targeting the dysregulated expression of these miRNAs or modulating gene activity may represent promising strategies for AML treatment. Further investigation into the functional roles of hsa-miRNAs in gene regulation and AML pathogenesis is needed to validate their potential as therapeutic targets or diagnostic biomarkers.

Here, for the first time, we identified that the changes in the association between some of the microRNAs and CD1a, CD2, and CD11b were the most apparent alterations among AML patients after infection with COVID-19. Some of these findings are aligned with and confirmed by the previous study of a systematic screening of genetic events in AML patients, which shows that the expression of CD markers CD1a, CD2, and CD11b in AML patients is significantly increased^39^. The present study is also consistent with the analysis of Li et al.^40^, which shows increased expression of CD markers CD1a, CD2, and CD11b in Mixed-phenotype acute leukaemia (MPAL) patients, and they have revealed that these three CD markers are among the CD markers that have the highest expression and have the most changes.

The present study evaluated the levels of various cytokines in AML using flow cytometry for diagnostic purposes. Flow cytometric immunophenotypic analysis was conducted on all patients. The expression levels of CD99, CD33, CD45, CD13, HLA-DR, CD64, CD11c, and CD117 ranged from 54.58% to 90.56% in all cases. Meanwhile, MPO, CD38, CD36, CD15, CD34, CD11b, and CD2 exhibited expression levels between 24.48% and 45.06%, with the remaining CD markers showing expression levels below 20% in all cases. These findings were consistent with previous studies on AML (Rasheed et al., 2021, Piñero et al., 2022). Previous researchers noted that markers such as MPO, CD13, CD33, CD15, and CD117 are specific to myeloid cells, while CD11c, CD64, CD14, and CD36 indicate monocytic lineage. Additionally, markers like CD19, CD22, CD10, CD79a, and CD20 are associated with B-lymphoid cells, while CD1a, CD2, CD3, CD4, CD5, CD7, and CD56 are linked to T-lymphoid/natural killer cells. Moreover, CD34, CD45, CD99, HLA-DR, Terminal deoxynucleotidyl transferase (TdT), and CD38 are commonly used as progenitor markers for diagnosing and classifying AML (Weir and Borowitz, 2001, Peters and Ansari, 2011).

Haematopoiesis is a highly regulated process controlled by complex molecular events that concurrently regulate hematopoietic stem cells’ commitment, differentiation, proliferation, and apoptosis. Substantial evidence now exists to demonstrate that microRNAs modulate haematopoiesis at the level of proliferation and differentiation and act as regulators of hematopoietic cell activity^41^. Differential expression of microRNAs in malignant cells compared to normal cells can be justified by their gene placement in cancer-related genomic regions, epigenetic mechanisms, and alterations in their processing mechanisms^42,43^. It has been established that variable expression of microRNAs plays a crucial role in leukemogenesis, and each cytogenetic alteration in AML is associated with the expression of a specific microRNA^9,44^. Generally, MicroRNA16 is a tumour suppressor that inhibits cell proliferation and induces apoptosis^45^. MicroRNA21 regulates immune responses in the body^46^, while MicroRNA125a, MicroRNA125b, and MicroRNA155 regulate hematopoietic stem cell activity^8^. MicroRNA192 plays a role in cell cycle arrest^47^, whereas MicroRNA221 induces cellular apoptosis^48^ and MicroRNA15a reduces apoptosis^49^. MicroRNA497 regulates cell proliferation^50^. In this study, the expression levels of these microRNAs were reported, and it was determined that they significantly impact disease progression. The increased expression levels of these microRNAs in AML patients are consistent with the findings of other studies that have reported higher expression of these microRNAs^51,52^. However, we presented for the first time the expression levels of microRNA and its correlation with CD markers in AML patients who were COVID-19 positive.

Considering that the expression patterns and levels of microRNAs can be used in disease prognosis and therapeutic responses^23^, the observed differences in the expression levels of microRNAs between AML patients and healthy individuals in this study are consistent with the results of other studies that have reported significant changes in the expression of microRNAs in various patients^28,53^. The decreased expression of microRNAs in the presence of CD markers and the concurrent decrease in CD marker expression in the presence of other CD markers align with the findings of studies by Shahrabi et al.^11^ and Gębarowska et al.^54^.

In a systematic review by Reyes-Long et al. (2023), they have demonstrated that microRNA expression levels differ between COVID-19 positive and negative patients, and these differences may be associated with the upregulation or downregulation of specific microRNAs^55^. Additionally, in the study by Shi et al. (2022), differences in microRNA expression among patients are observed^56^. The present study also found that the expression levels of microRNAs differed between COVID-19 positive and negative patients, and some microRNAs exhibited increased expression. The upregulation of microRNAs may not only be attributed to COVID-19 infection or vice versa but potentially due to the host's immune response to the virus^57^. Furthermore, considering the distinct expression levels of microRNAs in COVID-19 positive and negative patients, these changes in the expression levels of microRNAs could serve as suitable biomarkers^58,59^.

Identifying new types of microRNAs can be highly valuable and informative in understanding the mechanisms underlying various diseases, guiding therapeutic interventions, and promoting overall health improvement^60,61^. In the present study, microRNA 137 was identified as a novel biomarker involved in AML progression. MicroRNA137 plays a vital role in the evolution of the nervous system, the development of schizophrenia, and the maintenance of cellular homeostasis^62,63^. The present study showed an increased expression of MicroRNA137 in AML patients, while its expression was reduced in individuals who tested positive for COVID-19. However, a study by Wang et al. (2020) aimed to investigate the interaction between TRIM25 and MicroRNA137 and uncover their potential mechanisms in the progression of malignant AML. In that study, it was shown that there was a significant reduction in MicroRNA137 in blood samples of AML patients^3^. Cellular functional assays confirmed that the loss of MicroRNA137 substantially enhanced the invasion, migration, and proliferation abilities of AML cells. At the same time, overexpression of MicroRNA137 hindered the invasion, migration, and proliferation of AML cells. These findings indicated the tumour-suppressive role of MicroRNA137 in AML. However, further research is necessary due to the novelty of this microRNA. The precise significance and therapeutic target potential of MicroRNA137 may change as more is discovered about the molecular mechanisms behind AML and the involvement of microRNAs. Notably, research on MicroRNA137 and its role in AML is still underway. Targeting MicroRNA137in AML treatment may offer further therapeutic benefits, which will be further explored in subsequent research and clinical trials.

The present study demonstrated an increase in fold changes and microRNA expression in individuals who tested positive for COVID-19, and this increase was observed across all examined microRNAs. These findings are consistent with the results of studies conducted by Donyavi et al.^59^, Farr et al.^64^, and Li et al.^65^. This observation led us to understand that increased correlations for the expressions of genes in the cancer networks associated with decreased correlations for the expressions of genes in the standard networks might serve as valid biomarkers for early diagnosis of tumorigenesis and cancer progression. Finally, the identification of different MicroRNAs, the discovery of their target sequences, and the investigation of their gene expression pattern change in various cancers, including AML, along with their association with different cytogenetic abnormalities in AML patients, can provide a basis for more precise disease investigations, drug design, and the development of novel treatments for various types of cancer, including AML.

## Conclusions and future directions

The results showed that among AML patients, the expression of microRNAs increased, and the correlation between microRNAs led to their increased expression. Additionally, we demonstrated that the expression of microRNAs was significantly higher in individuals with a history of COVID-19 compared to those without a COVID-19 history. Remarkably, the expression level of MicroRNA137, a newly identified MicroRNA in this study, showed a significant difference between AML patients and healthy individuals, with higher expression observed in the presence of CD markers and FISH. These results shed light on the role of MicroRNA137 in the pathogenesis of AML.

The influence of COVID-19 in the interplay between CD marker and microRNA expression and gene mutations in AML provides a promising avenue for identifying novel therapeutic targets. Dysregulated microRNAs and the mutations they influence represent potential targets for therapeutic intervention in AML treatment. This study revealed that COVID-19 infection led to changes in the expression of specific miRNAs in AML patients. This highlights the importance of considering the effects of concurrent infections on disease progression and treatment outcomes in AML.

Additionally, this study identified miRNA137 as a novel AML biomarker, with significant expression differences between patients and healthy individuals. This highlights the potential of miRNA137 as a diagnostic or prognostic marker for AML. The strong correlation between miRNA137 and CD markers suggests potential crosstalk between miRNAs and cell surface markers in AML pathogenesis. Further exploration of these interactions could provide insights into disease mechanisms and therapeutic targets. Understanding the impact of COVID-19 on miRNA expression in AML patients leads to clinical decision-making and treatment strategies. Incorporating miRNA profiling into routine diagnostic and monitoring protocols may improve patient care and outcomes. Moreover, investigating the molecular mechanisms underlying the interactions between miRNAs, CD markers, and COVID-19 infection might lead to developing novel therapeutic approaches for AML management. Overall, exploring the microRNA expression in AML patients infected with COVID-19 opens new avenues for developing innovative and personalized treatments for AML patients.

### Supplementary Information

Supplementary Information.

## Data Availability

The raw data produced and analysed during the present study can be obtained from the corresponding author upon request, which is deemed reasonable.
