# Supplementary material for: The impact of COVID-19 on microRNA and CD marker expression in AML patients
Source: Sci Rep. 2024 Jun 20;14:14251. doi: 10.1038/s41598-024-64775-1 (PMC11190249; doi:10.1038/s41598-024-64775-1)
Supplement: Supplementary file 1 — Supplementary Information. [file 41598_2024_64775_MOESM1_ESM.pdf]

Supplementary Table 1:
List of antibodies in the flow cytometry panels used to detect CD immunophenotyping markers tested in this study.

Marker	Fluorophore	Clone	Catalog Number	Manufacturer
CD45	V500c	2D1	655873	BD Bioscience
CD19	PE-Cy7	SJ25C1	341113	BD Bioscience
cCD3	V450	UCHT1	560365	BD Bioscience
CD7	APC	M-T701	653311	BD Bioscience
CD38	APC-H7	HB7	656646	BD Bioscience
CD1a	HU APC	HI149	559775	BD Bioscience
CD99	PE	TÜ12	555689	BD Bioscience
CD5	PerCP-Cy5.5	L17F12	341109	BD Bioscience
CD14	APC-H7	MφP9	641394	BD Bioscience
MPO	FITC	5B8	333138	BD Bioscience
CD10	APC	HI10a	332777	BD Bioscience
CD117	PE-Cy7	104D2	339217	BD Bioscience
CD20	V450	L27	655872	BD Bioscience
CD34	PerCP-Cy5.5	8G12	347222	BD Bioscience
CD22	APC	S-HCL-1	333145	BD Bioscience
TdT	PE	E17-1519	332790	BD Bioscience
CD33	APC	P67.6	345800	BD Bioscience
CD13	PE	L138	347406	BD Bioscience
HLA-DR	V450	L243	655874	BD Bioscience
cCD22	APC	S-HCL-1	333145	BD Bioscience
CD2	APC	L303.1	335821	BD Bioscience
CD36	FITC	CLB-IVC7	656151	BD Bioscience
CD64	PE	10,1	644385	BD Bioscience
CD15	FITC	MMA	332778	BD Bioscience
CD11c	PerCP-Cy5.5	B-ly6	658330	BD Bioscience
CD11b	APC	D12	333143	BD Bioscience
cCD79a	PE	HM47	333152	BD Bioscience
CD3	V450	UCHT1	560365	BD Bioscience

Supplementary Figure 1:

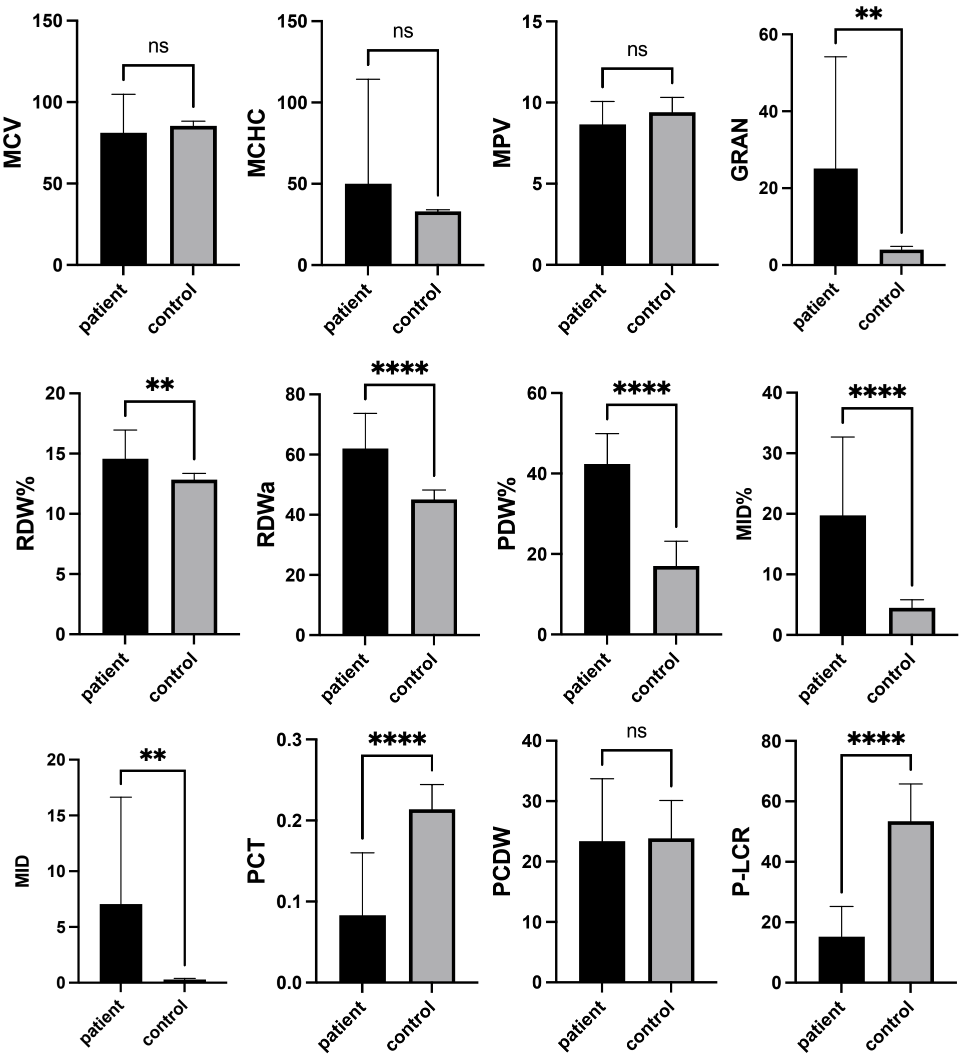

Supplementary Figure 1:
Complete Blood Count parameters in AML patients and healthy controls. MCV, Mean Cell Volume; MCHC, MCH Concentration; MPV, Mean Platelet Volume; GRAN, Granulocyte; RDW, Red Cell Distribution Width; MID, Intermediate cell count; PCT, Plateletcrit; PCDW, Platelet Component Distribution Width; P-LCR, Platelet Large Cell Ratio. *Statistically significant at 0.05 level.

Supplementary Figure 2:

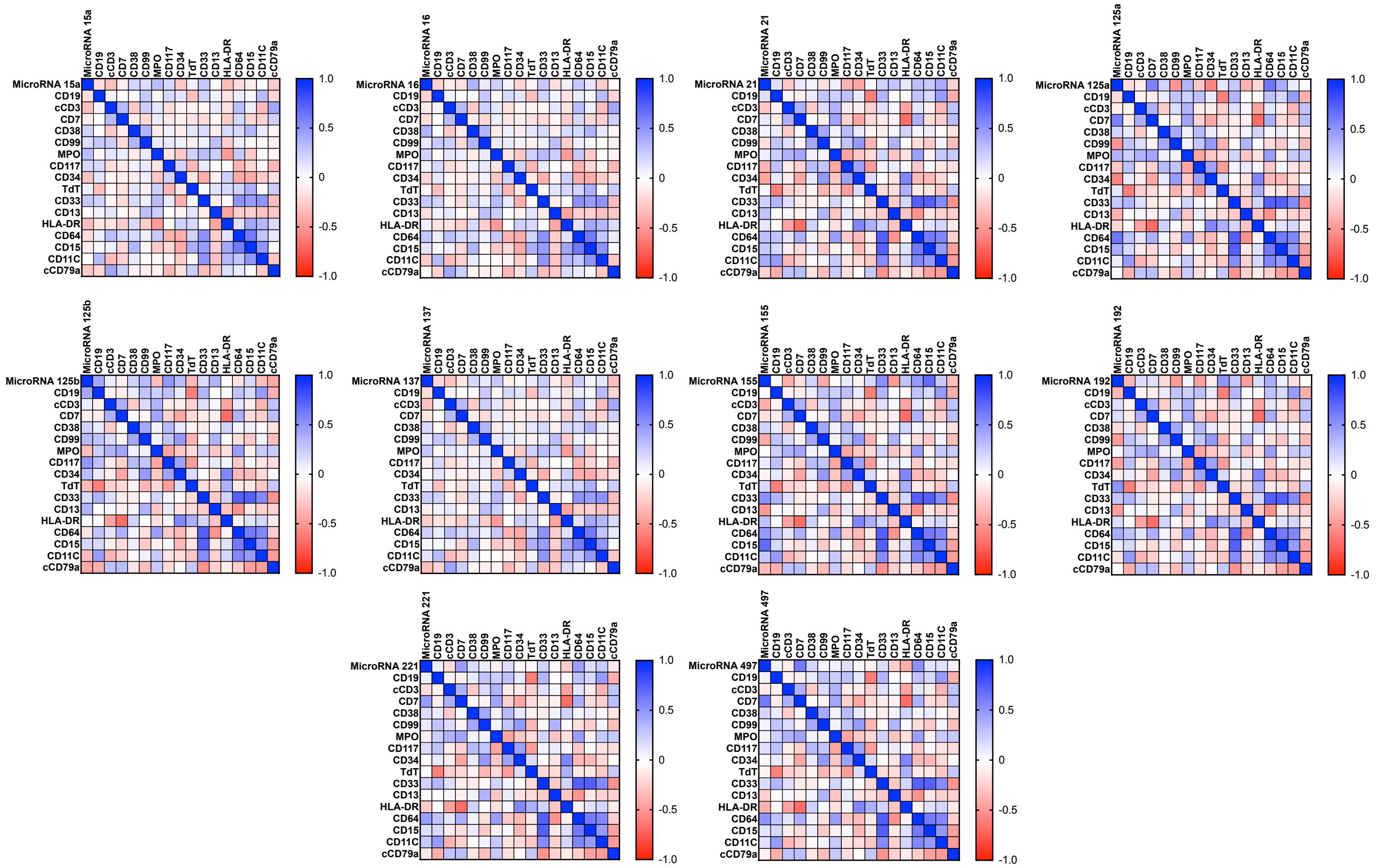

Supplementary Figure 2:
Assessment of microRNA expression and its correlation with CD markers. Spearman correlation coefficient analysis of microRNA expression levels with CD Markers.

Supplementary Figure 3:

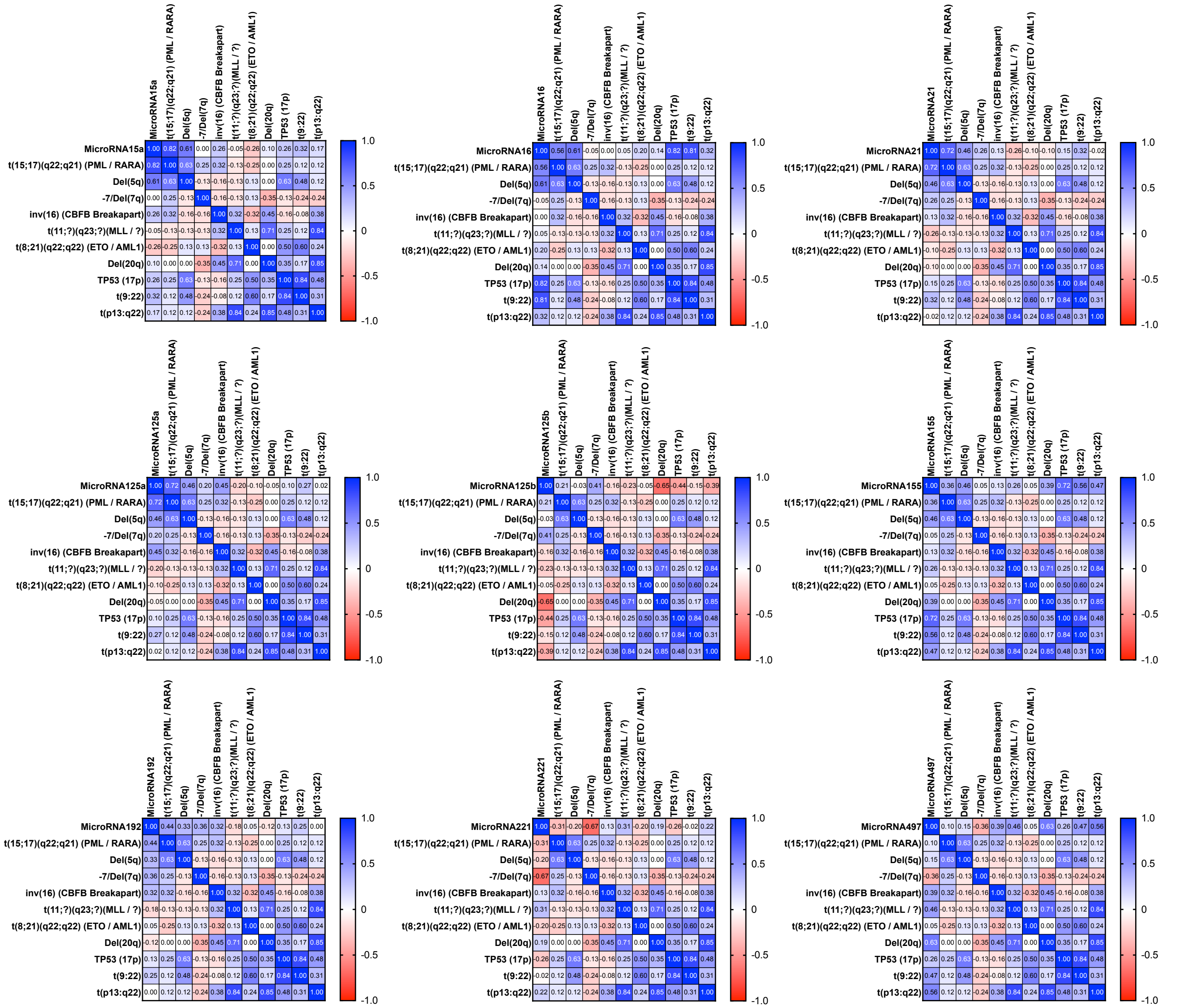

Supplementary Figure 3:

Assessment of microRNA expression and its correlation with CD markers. Spearman correlation coefficient analysis between each microRNA with FISH and genetic abnormalities ratios in AML patients.

.

Supplementary Figure 4:

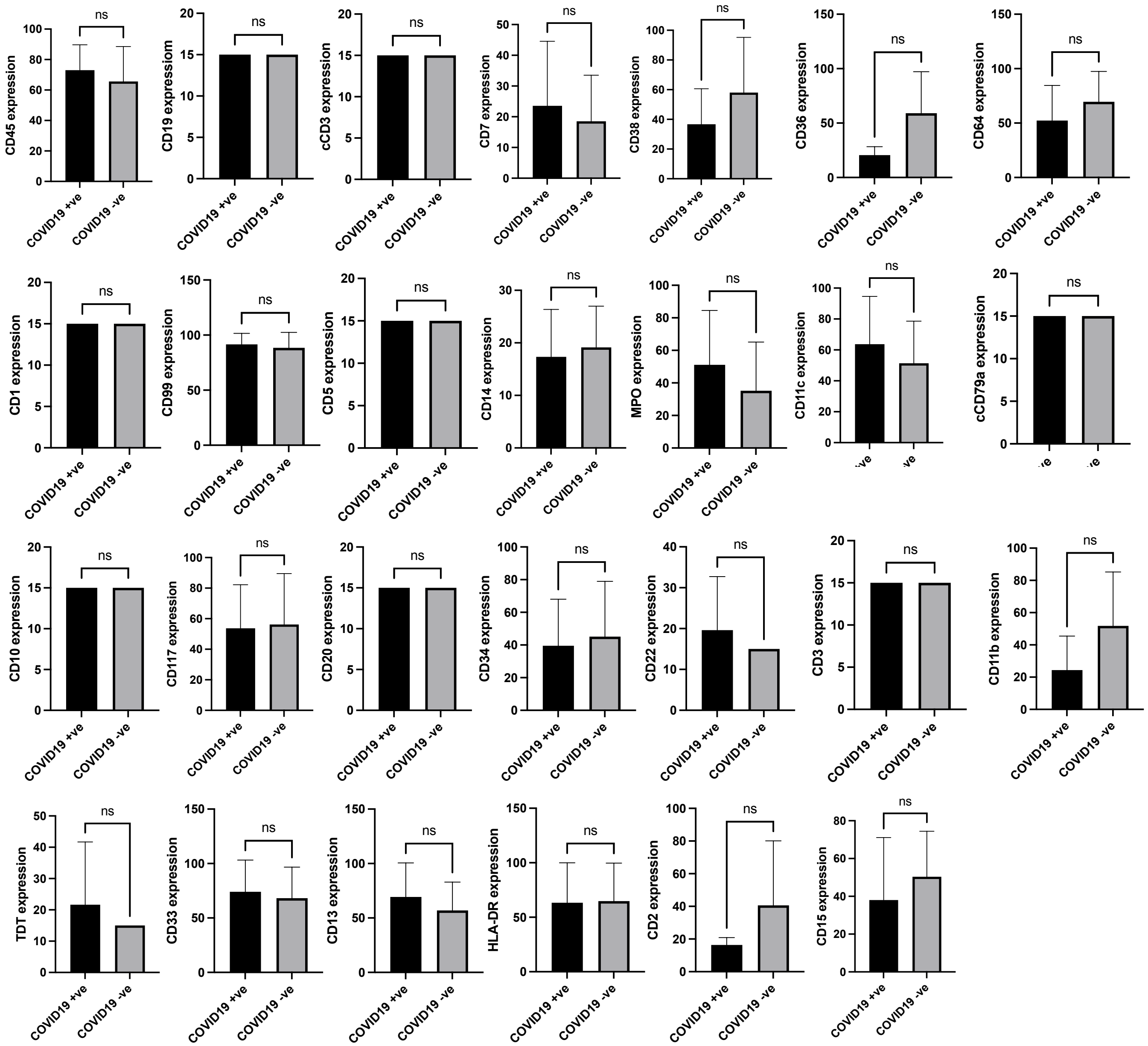

Supplementary Figure 4:
Analysis of CD immunophenotyping marker levels in AML patients concerning COVID-19.

Supplementary Figure 5:

A.

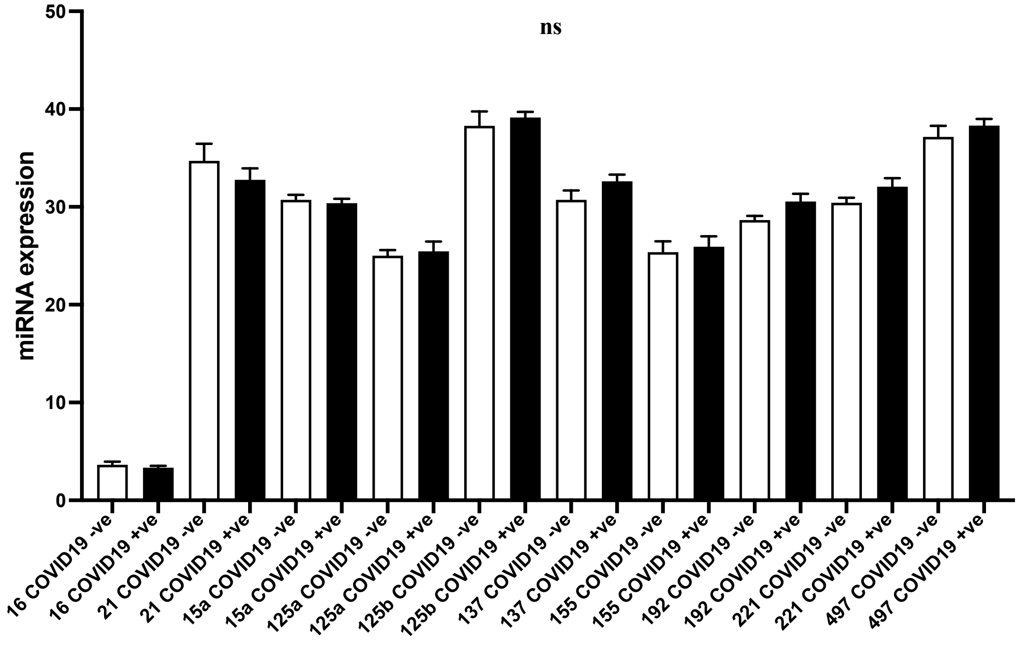

B.

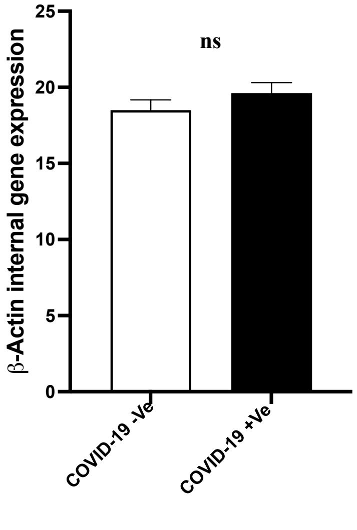

Supplementary Figure 5:
A. Analysis of microRNA expression levels in healthy control donors according to COVID-19. B. Histogram displaying the expression level of β-Actin, the internal control gene. ns, means statistically non-significant.
